# Auf dem Weg zur Demokratie – der Transformationsprozess 1990/91 und die Rolle der Gesellschaft für Psychiatrie und Nervenheilkunde in der DDR

**DOI:** 10.1007/s00115-023-01445-z

**Published:** 2023-02-20

**Authors:** Ekkehardt Kumbier, Kathleen Haack

**Affiliations:** 1 Arbeitsbereich Geschichte der Medizin, Universitätsmedizin Rostock, Doberaner Str. 140, 18057 Rostock, Deutschland; 2 Klinik und Poliklinik für Psychiatrie und Psychotherapie, Universitätsmedizin Greifswald, Greifswald, Deutschland Ellernholzstraße 1/2, 17489

**Keywords:** Psychiatriegeschichte, 20. Jahrhundert, Medizingeschichte, Ostdeutschland, Gesellschaft für Psychiatrie und Neurologie der DDR, History of psychiatry, 20th century, History of medicine, East Germany, Association for Neurology and Psychiatry in the GDR

## Abstract

**Ziel:**

Der Übergang von der sozialistischen Diktatur zu einer liberalen Demokratie in der DDR war mit politischen und gesellschaftlichen Umbrüchen verbunden. Die mit dem demokratisch-gesellschaftspolitischen Prozess einhergehende Transformation wird am Beispiel der Gesellschaft für Neurologie und Psychiatrie der DDR untersucht, die zur Vereinigung mit der Deutschen Gesellschaft für Psychiatrie und Nervenheilkunde (DGPN) führte.

**Methodik:**

Für die historische Untersuchung wurden Archivalien aus dem Archiv der DGPPN wie auch dem persönlichen Besitz damaliger Protagonisten genutzt und Zeitzeugeninterviews geführt.

**Ergebnisse:**

Der Transformationsprozess lässt sich auch für die Gesellschaft für Psychiatrie und Neurologie der DDR feststellen. Wie auf der politischen Ebene kam es 1990 auch auf der Vorstandsebene der Gesellschaft für Psychiatrie und Neurologie zu einem Legitimationsverlust. Das neue Demokratieverständnis erforderte die Beteiligung aller Mitglieder. Der Sprecherrat und die DGPN (Ost) waren zuständig für die Etablierung und Konsolidierung demokratischer Strukturen.

**Schlussfolgerung:**

Über den Transformationsprozess hinaus, ist bisher wenig über das Zusammenwachsen bekannt. Die Phase der Neuorientierung zu Beginn der 1990er-Jahre sollte für die DGPN ebenso untersucht werden wie die Frage nach dem Umgang mit dem vermuteten politischen Missbrauch der Psychiatrie in der DDR.

## Abstract (en)

**Objective:**

The transition from socialist dictatorship to liberal democracy in the GDR was associated with political and social upheaval. The transformation accompanying the democratic sociopolitical process is examined using the example of the Association for Neurology and Psychiatry of the GDR, which led to its unification with the German Association for Psychiatry and Neurology (DGPN).

**Method:**

For the historical investigation material from the archives of the DGPPN as well as the personal belongings of the protagonists of the time were used and eyewitness interviews were conducted.

**Results:**

The transformation process can also be seen for the Association for Neurology and Psychiatry of the GDR. As at the political level, there was also a loss of legitimacy at the board level of the Association for Psychiatry and Neurology in 1990. The new understanding of democracy required the participation of all members. The Spokesman Council and the DGPN (East) were responsible for establishing and consolidating democratic structures.

**Conclusion:**

Beyond the transformation process, little is known about the merger. The phase of reorientation at the beginning of the 1990s should be examined for the DGPN as well as the question of how to deal with the suspected political abuse of psychiatry in the GDR.

## Hintergrund

Für die Menschen in der Deutschen Demokratischen Republik (DDR) ging der Systemwechsel von der sozialistischen Diktatur zu einer liberalen Demokratie mit erheblichen Veränderungen in kurzer Zeit einher. Dieser war mit Umbrüchen von autoritären Gesinnungen hin zu bürgerlicher Eigenverantwortung verbunden, begleitet von demokratisch-gesellschaftspolitischen Prozessen. Wie solche – mit den Leitbegriffen der zeithistorischen Forschung formuliert – Transformationen bzw. Umbrüche im Kleinen aussahen, möchte der Beitrag am Beispiel der Auflösung der Gesellschaft für Psychiatrie und Neurologie der DDR, der Neugründung der Deutschen Gesellschaft für Psychiatrie und Nervenheilkunde in der DDR (DGPN Ost) und schließlich der Vereinigung mit der DGPN (West), nachzeichnen. Dabei erfolgt die Annäherung an das für die Psychiatriegeschichte bisher wenig vermessene Forschungsfeld der Transformationsprozesse um 1989/90 primär deskriptiv. Zum einen besteht die Notwendigkeit, die Vorgänge überhaupt und hier wohl erstmalig darzulegen. Zum anderen erlaubt die professionelle, mittlerweile auch zeitliche Distanz eine vorurteilsfreie Darstellung mit dem Versuch des Sich-Hineinversetzens in die Lage der Handelnden und Betroffenen (vgl. [1]).

Grundlage der historisch-kritischen Erörterungen bilden Unterlagen, die von den in der DGPN Ost und West aktiv Beteiligten zur Verfügung gestellt und mit denen Gespräche geführt wurden. Darüber hinaus konnten Archivalien aus der Zeit um 1990 im Archiv der Deutschen Gesellschaft für Psychiatrie und Psychotherapie, Psychosomatik und Nervenheilkunde (DGPPN) eingesehen werden.

## Das Ende der DDR und der Weg zur deutschen Einheit

Die Kettenreaktion von Ereignissen 1989/90 ließ das sozialistische Regime in der DDR innerhalb kurzer Zeit kollabieren. Bereits im März 1990 fand die erste freie Wahl der noch bestehenden DDR statt. Sie führte zur Bildung einer Regierung, die die Überreste der SED-Diktatur überwinden und den Übergang zur westlichen Demokratie vollziehen sollte. Damit waren die politischen Voraussetzungen geschaffen worden, um mit der Bundesregierung möglichst rasch über die Wiedervereinigung zu verhandeln, die Anfang Juli mit den Gesprächen über den Einigungsvertrag konkret wurden. Auf dem Weg zum vereinten Deutschland mussten neue Strukturen geschaffen und alte beseitigt werden. Schließlich erfolgte auf der Grundlage des Einigungsvertrages am 03.10.1990 die deutsche Wiedervereinigung durch den Beitritt der DDR zur BRD (vgl. [10, 14]).

Die folgende tiefgreifende Umgestaltung brachte enorme Herausforderungen mit sich. So gab es hinsichtlich der Verantwortlichen in führenden Positionen unterschiedliche Auffassungen. Einige forderten, dass Führungskräfte entlassen werden sollten, die im SED-System in leitenden Positionen tätig gewesen waren und Verantwortung getragen hatten. So kam es zu Beginn der 1990er-Jahre zu einem breiten Austausch von Führungskräften in verschiedenen gesellschaftlichen Bereichen (vgl. [5]). Für einige Ostdeutsche erwiesen sich die zu DDR-Zeiten getroffenen Lebensentscheidungen unter den neuen Bedingungen als nachteilig. Einige erlebten eine Entwertung ihrer bisherigen Leistungen und fühlten sich als Verlierer. Für andere wiederum eröffneten sich in der Zeit des Aufbruchs neue Perspektiven (vgl. [3]). Prinzipiell wurde im Einigungsprozess eine Überführung in die westdeutschen Strukturen angestrebt, nach einer Angleichung und weniger nach einer gleichberechtigten Vereinigung auf Augenhöhe. Dieses Streben dominierte in dieser Zeit und war nicht zuletzt der Wunsch vieler Ostdeutschen selbst.

## Der Übergang: die Gesellschaft für Psychiatrie und Nervenheilkunde in der DDR

Unmittelbar vor der letzten Gesamttagung der Gesellschaft für Psychiatrie und Neurologie der DDR (07. bis 09.02.1990) in Leipzig, hatte dort vom 5. bis 7. Februar auch der 1. Kongress der Psychiatrie der DDR stattgefunden. Er war von der Sektion Psychiatrie, die um diese Zeit ca. 400 Mitglieder hatte^1^, organisiert worden. In der Mitgliederversammlung am 06.02.1990 standen die Konstitution einer Gesellschaft für Psychiatrie der DDR (in Nachfolge der bisherigen Sektion Psychiatrie) mit der Neuwahl des Vorstandes an. Dem Vorstand der Sektion gehörten seit der letzten Wahl im November 1983 als Vorsitzender Klaus Ernst (Rostock) sowie Cornelia Hirsch (Leipzig), Diether-Rudolf Burian (Neuruppin), Christian Donalies (Wittstock), Günter Hoffmann (Hubertusburg), Hugo von Keyserlingk (Schwerin), Helmut Kulawik (Berlin), Ehrig Lange (Dresden), Siegfried Schirmer (Brandenburg), Helmut Späte (Halle) und Klaus Weise (Leipzig) an. Nach einer regen Diskussion wurde unter dem Eindruck der gesellschaftlichen Veränderungen keine neue Gesellschaft gebildet und auch kein Vorstand gewählt, sondern ein Sprecherrat der weiterbestehenden Sektion Psychiatrie gebildet. Dessen Aufgabe sollte die Einleitung eines Demokratisierungsprozesses innerhalb der Fachgesellschaft sein. Die Initiative ging spontan von den Mitgliedern aus und entsprach dem Wunsch nach Veränderung und mehr Mitbestimmung^2^^,^^3^. Der westdeutsche Kinderpsychiater Helmut Remschmidt berichtete der DGPN (West) unmittelbar nach seiner Teilnahme, dass „einige bisher an führender Stelle tätigen Psychiater sehr angegriffen wurden“ und empfahl aufgrund der „Dynamik in der ganzen Entwicklung […] ganz dringend“ Kontakt zum Sprecherrat aufzunehmen [17]. Diesem gehörten Erdmuthe Fikentscher (Halle/S.), Heinz Benkenstein (Hildburghausen) und Klaus-Dieter Waldmann (Plauen) an, aber auch die beiden bisherigen Vorstandsmitglieder Ernst und von Keyserlingk. Bereits wenige Tage später informierte der Sprecherrat in einer Presseerklärung über seine freie Wahl und verwies darauf, dass „Jahrzehntelang […] dieses Fachgebiet durch die zuständigen Leitungsgremien bevormundet, vernachlässigt und mit folgenschweren Tabus und Verboten belegt“ [17] worden sei. Der Sprecherrat musste in der Übergangsphase von der alten zur neuen Ordnung aber nach vorn schauen. Er sollte die Gründung einer Gesellschaft für Psychiatrie und Nervenheilkunde in der DDR mit dem Ziel der Vereinigung mit der westdeutschen DGPN vorbereiten. Die „etablierten“ DDR-Psychiater, die in Leitungsfunktionen bisher die Geschicke der DDR-Fachgesellschaft bestimmt hatten, waren hingegen an einem sofortigen Zusammenschluss interessiert. Sie spekulierten offenbar, dass sie als bekannte Fachvertreter in den Vorstand aufgenommen werden würden. Nach ersten Kontakten trat der Vorstand der DGPN (West) schließlich im Sommer 1990 an den Sprecherrat mit der Frage nach der Vereinigung beider Fachgesellschaften heran und bat um entsprechende Unterstützung^4^^,^^5^.

**Figure 1:**
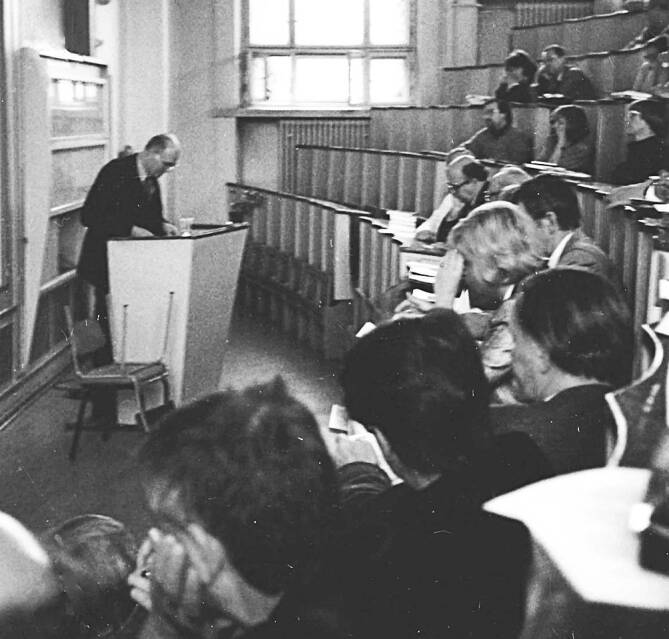

Zunächst wurde am 09.06.1990 auf einer Vollversammlung der Mitglieder der Sektion Psychiatrie in Berlin die Gesellschaft für Psychiatrie und Nervenheilkunde in der DDR (DGPN Ost) gegründet (Abb. 1). Vertreter des „alten“ Vorstandes nahmen daran nicht teil, sodass im Prinzip nun zwei psychiatrische Fachgesellschaften in der DDR existierten^6^ [15]. Zu dieser Gründungssitzung war auch der Präsident der westdeutschen DGPN, Johannes Meyer-Lindenberg, eingeladen worden, der eine „Stellungnahme im Stile ‚kritischer Sympathie‘ mit der Hoffnung auf ‚vernunftsbezogene‘ Annäherung“ verlas [18]. Bereits die Namensgebung der neuen Fachgesellschaft mit dem Zusatz „in der DDR“ verdeutlicht die Absicht, sich von der bisherigen DDR-Gesellschaft abzugrenzen. Für die Neuwahl eines Vorstandes hatte der Sprecherrat die Regionalgesellschaften um Beteiligung gebeten. Diese sollten jeweils zwei Kandidaten vorschlagen, um eine „basisdemokratische Wahl“ zu ermöglichen [19]. Ziel war, geeignete Fachvertreter zu finden, die nicht in die staatliche Ideologie verstrickt gewesen und mehrheitsfähig waren. Das war erforderlich, um mit dem Vorstand der DGPN (West) über die Vereinigung sprechen zu können. Die bisherigen Führungskräfte hatten nicht mehr das Vertrauen aller Mitglieder, galten als „staatsorientierte Psychiater“^7^^,^^8^. In diesem Zusammenhang werden immer wieder Bernd Nickel und Jochen Neumann genannt, die auch im Vorstand der Gesellschaft für Psychiatrie und Neurologie der DDR aktiv waren. Nickel war u. a. ärztlicher Leiter des Wilhelm-Griesinger-Krankenhauses in Berlin. Er hatte die inoffizielle Zusammenarbeit mit dem MfS im Gegensatz zu Neumann verweigert [12, S. 230]. Neumann war u. a. ärztlicher Direktor des Wilhelm-Griesinger-Krankenhauses Berlin, ordentlicher Professor für Psychiatrie an der Friedrich-Schiller-Universität Jena und von 1983 bis 1989 Generaldirektor des Deutschen Hygiene-Museums in Dresden. Er unterhielt regelmäßig inoffizielle Verbindungen zum MfS [12, S. 621ff.]. Neumann und Nickel waren beide „Reisekader“ und vom MfS als politisch zuverlässig eingeschätzt worden [12, S. 651].

Die westdeutschen Psychiater begegneten ihren Kollegen aus dem Osten überwiegend offen, teils aber auch mit Zurückhaltung und Vorsicht. Das betraf auch Gert-Eberhard Kühne als letzten Vorsitzenden der Gesellschaft für Psychiatrie und Neurologie der DDR, dem einige westdeutsche Psychiater zuvor bei internationalen Kongressen begegnet waren und den sie als Repräsentanten der DDR wahrgenommen hatten^9^^,^^10^. Nach der Wahl konnte sich der neue Vorstand der DGPN (Ost) mit Otto Bach (Vorsitzender), Klaus-Dieter Waldmann und Bärbel Schliack (Stellvertreter), Joachim Morgner (Sekretär) und Georg Bonitz (Schatzmeister) sowie Werner Felber und Klaus Ernst konstituieren. Er war während der Gründungsveranstaltung in Berlin frei und demokratisch gewählt worden, worauf in dieser Zeit besonders geachtet worden sei^11^.

**Figure 2:**
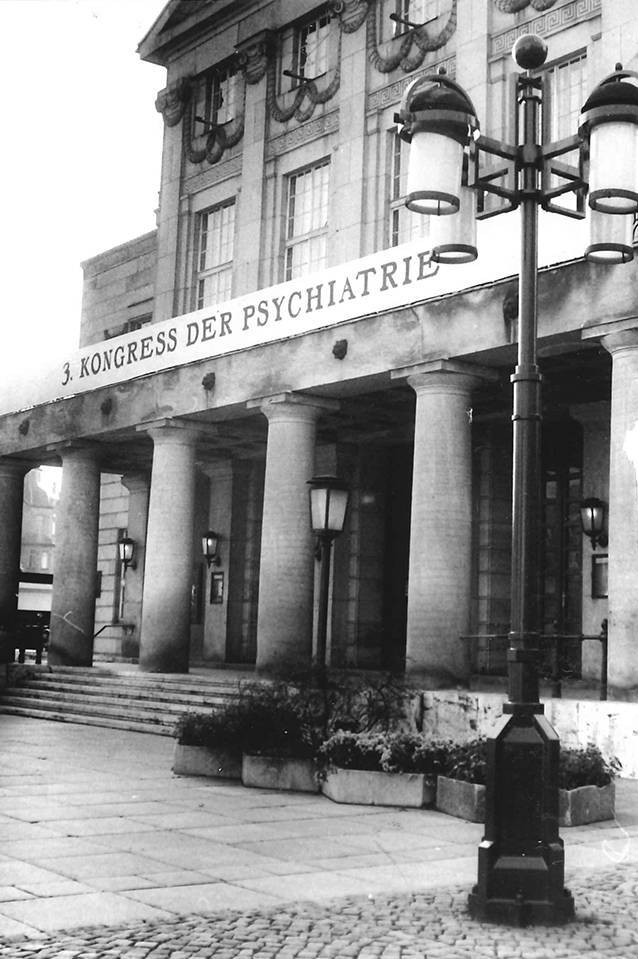

Als besondere Wegmarke kann der „3. Kongreß für Psychiatrie“ am 12.11.1990 in Weimar (Abb. 2) angesehen werden. Dieser war noch von der „alten“ Gesellschaft für Psychiatrie und Neurologie der DDR organisiert worden. Deren Auflösung war bereits am 07.02.1990 in einer Gemeinschaftssitzung aller Sektionen (Psychiatrie, Neurologie, medizinische Psychologie und Neuropsychiatrie des Kindes- und Jugendalters) während der letzten Gesamttagung in Leipzig beschlossen worden. Der Vorstand der „neuen“ DGPN (Ost) hatte die „Bemühungen des Kongreßpräsidenten [Gert-Eberhard Kühne, d. V.], die neue Gesellschaft [DGPN (Ost)] in Rechtsnachfolge der früheren Gesellschaft für Psychiatrie und Neurologie zu sehen“ als falsch zurückgewiesen und betont, dass „die neue DGPN (DDR) […] nicht mit der Veranstaltung identifiziert werden [will]“ [20]. Der „Kongreß [musste sich] eindeutig auf die frühere DDR-Gesellschaft für Neurologie und Psychiatrie“ beziehen [21]. Der „alte“ Vorstand der Gesellschaft für Psychiatrie und Neurologie der DDR musste sich fügen und hatte somit erkennbar an Einfluss verloren.

## Die Vereinigung: die gesamtdeutsche Gesellschaft für Psychiatrie und Nervenheilkunde

In den Händen der Vorstandsmitglieder der DGPN (Ost) lag nun die Aufgabe, die Vereinigung mit der DGPN (West) zu einer gesamtdeutschen DGPN vorzubereiten und den Beitritt der ostdeutschen Mitglieder zu ermöglichen. Bis Ende 1990 fanden intensive Gespräche statt, auch mit Vertretern der niedergelassenen Nervenärzte und hier vor allem mit dem Berufsverbandverband Deutscher Nervenärzte (BVDN). Von Seiten der DGPN (West) waren insbesondere der Präsident, Meyer-Lindenberg, und der Vizepräsident, Eberhard Lungerhausen, und der Vorsitzende des BVDN, Hans-Adolf Paul, beteiligt. Nach dem Tod Meyer-Lindenbergs im Februar 1991 übernahm Uwe Henrik Peters diese Aufgabe.

Mit der Vereinigung wurde die DGPN (Ost) am 03.07.1991 in Dresden aufgelöst. Sie hatte ihre Funktion erfüllt. Viele Mitglieder traten in die gesamtdeutsche DGPN ein oder fanden sich in den regionalen Gesellschaften wieder. In den Vorstand der gesamtdeutschen DGPN wurden Otto Bach, Klaus Ernst, Ehrig Lange und Bärbel Schliack für eine Übergangszeit von zwei Jahren kooptiert [21]. Der zu diesem Zeitpunkt fast 70-jährige Lange bat im Juni 1991 aufgrund seines Lebensalters um die vorzeitige Beendigung seiner „Tätigkeit als assoziiertes Vorstandsmitglied“ [22]. Inwieweit für diese Entscheidung seine Zusammenarbeit mit dem Ministerium für Staatssicherheit der DDR ausschlaggebend war (vgl. [9, S. 611–615]) ist bisher nicht bekannt.

Die dynamische Entwicklung hin zu einer gesamtdeutschen DGPN rief auch kritische Stimmen hervor. Einige, vor allem die aus der DDR in die BRD geflüchteten Psychiater, standen einer ungeprüften Aufnahme aller Mitglieder der DDR-Fachgesellschaft kritisch, bisweilen sogar ablehnend gegenüber. Ein Grund war, dass zu dieser Zeit ein systematischer politischer Missbrauch der Psychiatrie in der DDR vermutet wurde^12^. Einige Vertreter der Bundesdirektorenkonferenz warnten „vor zu schneller Verbrüderung oder Assoziationen“ und betonten, dass eine Vereinigung erst erfolgen solle, „wenn ein Selbstreinigungsprozeß in der DDR begonnen hat und insbesondere erst dann, wenn nicht die alten führenden sog. Reisekader wieder an der Spitze der Gesellschaft sitzen und dann auf einmal bei uns auch das große Wort führen“, so der Ärztliche Direktor des Psychiatrischen Krankenhauses Eichberg, Steffen Haas, in einem Schreiben vom 13.06.1990 an die DGPN (West; [22]). Dem Verband leitender Ärztinnen und Ärzte der Kliniken für Psychiatrie und Psychotherapie gehörten mit Steffen Haas, Ulrich Trenckmann und Fritz Reimer (Vorsitzender von 1987 bis 1992) einflussreiche Fachvertreter an, die aus der DDR stammten und die die Entwicklung hin zur Vereinigung besonders kritisch verfolgten. Sie hatten schon im Mai 1990 den Präsidenten der DGPN (West), Meyer-Lindenberg, nach ersten Kontakten zu Repräsentanten der DDR-Psychiatrie im Rahmen der Bundesdirektorenkonferenz im April 1990 gebeten, nicht mit den ehemaligen „Reisekadern“ zu verhandeln, sondern diese zur eigenen Vergangenheitsbewältigung aufzufordern und vor allem geeignete Vertreter zu finden, um weitere Schritte zu besprechen [17]. Vor diesem Hintergrund hatte sich der erweiterte Vorstand der DGPN (West) noch am 08.06.1990, einen Tag vor Gründung der ostdeutschen DGPN, darauf verständigt, dass „man sich bedeckt und abwartend verhalten […], aber sich zur Verfügung stellen sollte, bis der nötige Klärungsprozeß der DDR abgeschlossen ist“. Gleichzeitig wurde festgestellt: „Die Institutionalisierung einer demokratisch gewählten Gesellschaft für Neurologie und Psychiatrie ist noch keineswegs gesichert“ [18].

Auch vor diesem Hintergrund war die Frage nach der Aufnahme der Mitglieder der DDR-Fachgesellschaft ein Balanceakt. Einerseits ging es darum, ein berechtigtes Maß an Kontrolle sicherzustellen, andererseits aber auch die Offenheit der nun gesamtdeutschen DGPN zu repräsentieren. Der Vorstand der DGPN (West) hatte im September 1990 beschlossen, dass es keinen kumulativen Beitritt der DDR-Gesellschaft zur DGPN geben solle, sondern nur Einzelbewerbungen möglich sind [18]. Die Mitglieder der DDR-Gesellschaft  konnten gemäß der Satzung auf Antrag hin und unter Benennung von zwei Bürgen, die bereits Mitglieder waren, aufgenommen werden. Für Neumitglieder aus der DDR galt bis zur Vorstandswahl (1992) ein reduzierter Mitgliedsbeitragssatz [21]. Aber schon der Begriff des Bürgen implizierte in dieser sensiblen Zeit für einige eine Bevormundung, sodass Uwe Henrik Peters im Februar 1991 in einem Brief an Otto Bach klarstellen musste, dass dem Antrag „2 Referenzen – nicht Bürgen!“ beigefügt werden sollen und hinzufügte: „Es ist aber meine große Hoffnung, diese Phase des Mißtrauens so rasch wie möglich und mit ihrer Hilfe zu überwinden“ [18].

## Diskussion

Der politische Systemwechsel und Prozess der Wiedervereinigung führten nicht nur auf der staatlichen Ebene zu einschneidenden Veränderungen, sondern in allen gesellschaftlichen Bereichen. Die Transformation im Gesundheitswesen der DDR führte im Prozess der deutschen Vereinigung zu einer raschen und kompromisslosen Abwicklung der ostdeutschen Gegebenheiten (vgl. [7]). Neben einer Dominanz bundesdeutscher Akteure fehlten in diesem gesundheitspolitischen Entscheidungsprozess autonome gesellschaftliche Akteure in der DDR. Das westdeutsche Gesundheitssystem wurde weitgehend übernommen, das ostdeutsche wegen seiner Einbindung in das Staats- und Gesellschaftssystem mit der Durchsetzung gesundheitspolitischer Ziele von Staat und Partei hingegen kritisiert (vgl. [13]). Die mit der Transformation verbundenen Veränderungen wurden auf verschiedenen Ebenen untersucht. Auf der Makroebene zeigt sich, dass die Prozesse der politischen Steuerung des Medizinsystems durch neue gesetzliche und ökonomische Anreizstrukturen und auch Qualitätsstandards zur Evaluierung der Gesundheitseinrichtungen durchgesetzt wurden. Auf der Mesoebene sind etwa veränderte Karrierepolitik oder Arbeitsbeziehungen und -formen wie die Förderung der ambulanten Patientenversorgung durch Arztpraxen erkennbar. Auf der Mikroebene finden sich konkrete berufliche Brüche und Neuorientierungen der Ärzte und Ärztinnen (vgl. [9, 11]).

Vergleichsweise wenig ist bisher über die Transformation der ostdeutschen Psychiatrie bekannt. In einigen Veröffentlichungen werden die Veränderungen in bestimmten Regionen oder von ausgewählten Aspekten der psychiatrischen und auch kinderpsychiatrischen Versorgung beschrieben. Das betrifft beispielsweise die Verweildauer von Patientinnen und Patienten mit psychischen Erkrankungen in stationären Einrichtungen, die Häufigkeit ihrer Institutionalisierung, die Verringerung der psychiatrischen Bettenkapazität, neue Formen der Behandlung und Betreuung und die Unterbringung psychisch kranker Straftäter (vgl. [4, 8]).

In Interviews erinnern sich rückblickend ehemalige Mitarbeiter der Kliniken, welche Auswirkungen der Transformationsprozess und die geforderte Aufarbeitung der Vergangenheit nach 1990 auf das Erinnern an die sog. DDR-Psychiatrie und deren Reform hatten [2].

Wie in dieser Arbeit gezeigt werden konnte, lässt sich der mit der Wiedervereinigung verbundene Transformationsprozess auch für die Gesellschaft für Psychiatrie und Neurologie der DDR feststellen: Auf der Vorstandsebene kam es 1990 zu einem Legitimationsverlust. Das neu erwachsene Verständnis von Demokratie erforderte die Beteiligung möglichst aller Mitglieder inklusive der Regionalgesellschaften. Der Sprecherrat und die DGPN (Ost) erfüllten als „Übergangs-Institutionen“ eine wichtige Funktion. Sie waren im Prozess der Demokratisierung zuständig für die Etablierung demokratischer Strukturen, um diese schließlich durch die Vereinigung konsolidieren zu können. Die bis dahin unabhängig voneinander existierenden Fachgesellschaften für Neurologie und Psychiatrie vereinigten sich in organisatorischer Hinsicht. Doch bedeutete das 1990 wie die staatliche Wiedervereinigung eben auch, dass zwei deutsche Teilgesellschaften mit ihrer zwar nie voneinander losgelösten, im Wesentlichen aber doch von Unterschieden geprägten Geschichte nun wieder zusammenfinden mussten. 40 Jahre Demokratie auf der einen und 40 Jahre SED-Diktatur auf der anderen Seite hatten beide Teilgesellschaften auch mental nachhaltig geprägt. Das zeigt etwa die zu Beginn der 1990er-Jahre geführte Auseinandersetzung um die Institution Psychiatrie und vor allem ihre Einbindung in das Herrschaftssystem, die primär öffentlich und sehr emotional geführt wurde (vgl. [6]). Das mag die Annäherung zwischen ost- und westdeutschen Fachvertretern anfangs erschwert haben. Erst später folgte eine sachliche Auseinandersetzung mit einer zunehmend differenzierten Betrachtungsweise. Die deutsche Wiedervereinigung war und ist eine gesamtgesellschaftliche Aufgabe, die Menschen zusammenführte, die ganz unterschiedlich sozialisiert worden waren und dementsprechend verschiedene Wertvorstellungen hatten. Somit war die Wiedervereinigung sowohl im Großen wie im Kleinen nicht nur von objektiven Problemen, sondern auch von subjektiven Verständigungsschwierigkeiten geprägt (vgl. [3]). Im Bereich der Fachgesellschaften muss das noch herausgearbeitet werden, denn trotz aller fachlichen Gemeinsamkeiten empfanden einige Mitglieder der ehemaligen DDR-Fachgesellschaft den Vereinigungsprozess möglicherweise als oktroyiert. Dabei sollte berücksichtigt werden, dass Menschen, die bis dato in der DDR gelebt hatten, gezwungen wurden, ihre gewohnten Lebensgleise zu verlassen, möglicherweise verunsichert waren und ein Stück Identität verloren. Sie empfanden Nöte, die ihre westdeutschen Kollegen so nicht oder anderes empfanden. Gleichzeitig war verständlich, dass westdeutsche Kollegen eine gewisse Skepsis der politischen Verstrickungen, aber auch der im Raum stehenden Missbrauchsvorwürfe wegen empfanden. Über den weiteren Prozess des Zusammenwachsens wissen wir wenig. Bisher ist nicht untersucht, ob und wie sich die DGPN zu Beginn der 1990er-Jahre in der Phase der Neuorientierung ausrichtete, inhaltlich neue Schwerpunkte formuliert wurden und schließlich, wie dem vermuteten politischen Missbrauch der Psychiatrie in der DDR begegnet wurde.

## Footnotes

1 Vgl. zu den nachfolgenden Angaben den Vorlass von Prof. Dr. Klaus Ernst, Ordner „DGPN-Ost“, der den Zeitraum zwischen Februar 1990 und Dezember 1991 umfasst, unpag. [16].

2 Fikentscher E (2021) Persönliche Mitteilung am 13.04.2021.

3 Waldmann K‑D (2021) Persönliche Mitteilung am 14.04.2021.

4 Fikentscher E (2021) Persönliche Mitteilung am 13.04.2021.

5 Waldmann K‑D (2021) Persönliche Mitteilung am 14.04.2021.

6 Felber W (2021) Persönliche Mitteilung am 31.03.2021.

7 Bach O (2021) Persönliche Mitteilung am 06.04.2021.

8 Fikentscher E (2021) Persönliche Mitteilung am 13.04.2021.

9 Häfner H (2021) Persönliche Mitteilung am 06.09.2021.

10 Peters UH (2021) Persönliche Mitteilung am 18.08.2021.

11 Felber W (2021) Persönliche Mitteilung am 31.03.2021.

12 Felber W (2021) Persönliche Mitteilung am 31.03.2021.
